# Evaluation of the antioxidant, anti-inflammatory, and anti-tumoral properties of bioactive compounds extracted from murta berries (*Ugni molinae* T.) dried by different methods

**DOI:** 10.3389/fpls.2023.1095179

**Published:** 2023-05-18

**Authors:** Jéssica López, Antonio Vega-Gálvez, Kong S. Ah-Hen, Angela Rodríguez, Issis Quispe-Fuentes, Carla Delporte, Gabriela Valenzuela-Barra, Yennyfer Arancibia, Angara Zambrano

**Affiliations:** ^1^ Escuela de Alimentos, Pontificia Universidad Católica de Valparaíso, Valparaíso, Chile; ^2^ Departamento de Ingeniería en Alimentos, Universidad de La Serena, La Serena, Chile; ^3^ Instituto de Ciencia y Tecnología de los Alimentos, Universidad Austral de Chile, Valdivia, Chile; ^4^ Instituto Multidisciplinario de Investigación y Postgrado, Universidad de La Serena, La Serena, Chile; ^5^ Laboratorio de Productos Naturales, Facultad de Ciencias Químicas y Farmacéuticas, Universidad de Chile, Santiago, Chile; ^6^ Instituto de Bioquímica y Microbiología, Facultad de Ciencias, Universidad Austral de Chile, Valdivia, Chile; ^7^ Center for Interdisciplinary Studies on the Nervous System (CISNe), Universidad Austral de Chile, Valdivia, Chile

**Keywords:** anti-inflammatory activity, anti-tumoral activity, antioxidant potential, drying methods, murta fruits, phenolic compounds

## Abstract

This study evaluated the effects of different drying methods (freeze drying, vacuum drying, infrared drying, convective drying, and sun drying) on the biological properties of berries from the Chilean murta (*Ugni molinae* Turcz) shrub. Physical-chemical properties (proximal composition, dietary fiber, sugars) were determined. Total phenolic content through the method of Folin-Ciocalteau, the profile of phenol compounds was determined by HPLC, and antioxidant potential by DPPH and ORAC assays were also evaluated. The topic anti-inflammatory effect was evaluated by mice´s ear edema, and *in vitro* anti-tumoral activity was tested by MTT assay. The chemical properties of dried berries differed significantly based on the drying method: freeze-dried murta berries showed increased total phenolic content extracted over fresh and dried samples. In addition, this lyophilized extract stood out in its antioxidant potential, in both assays evaluated (DPPH and ORAC), compared to the other drying methods. Notwithstanding, vacuum- and infrared-dried murta also showed a higher ORAC value. Antioxidant potential was significantly associated with phenolic compounds catechin and pyrogallol, which were the most abundant phenolic compounds present in all samples. The anti-inflammatory activity was most effective under freeze-drying and vacuumdrying conditions. Moreover, vacuum drying and infrared drying best preserved the anti-tumoral effect on cancer cells.

## 1 Introduction

Great interest in functional foods—or those that may protect human health—has extended to studies on edible berries in Chile, including meli (*Amomyrtus meli*), maqui (*Aristotelia chilensis*), chequén (*Luma chequen*), arrayán (*Luma apiculata*), calafate (*Berberis microphylla*), and murta (*Ugni molinae* T.). Of particular interest are their potential as a source of vitamins, minerals, fiber, and antioxidants and the fact that they could be used as ingredients in functional foods (Ruiz et al., 2010; Ramirez et al., 2015; Brauch et al., 2016; Rodríguez et al., 2016). Murta is the most well-known Myrtaceae native growing in south-central regions of Chile, specifically in the Coast Mountain and the pre-Andean mountains between the Maule and Aysén regions, including the Archipelago of Juan Fernández (Castro et al., 2021), which produces a small globular berry with a pleasant taste and smell that yields high and diversified bioactive phenolic compounds (Augusto et al., 2014; Junqueira-Gonçalves et al., 2015), as free and bound (Rodríguez et al., 2014); indeed, previous profiles have found flavonols, phenolic acids, flavanols, and anthocyanins (Alfaro et al., 2014; Augusto et al., 2014; Junqueira-Gonçalves et al., 2015).

While most studies of these phenolic compounds have been extensively focused on antioxidant capacity and, in murta fruits, on phytochemical composition and *in vitro* antioxidant activity, other biological activities remain to be explored. Indeed, the anti-inflammatory activity of only murta leaves has been described, attributed to the presence of triterpenoid acids, including 2-α-hydroxy derivatives alphitolic, corosolic, and asiatic acids (Delporte et al., 2007; Goity et al., 2013; Otero et al., 2023). Furthermore, while berry extracts have been shown to inhibit cancer cell proliferation *in vitro* (Golovinskaia and Wang, 2021; Bouyahya et al., 2022; Mazzoni et al., 2019), these also contain a wide range of phenolic compounds such as delphinidin-3-rutinoside, chlorogenic acid, and epicatechin that can inhibit carcinogenesis and prevent oxidative stress (Miladinovic et al., 2023).

The drying appears suitable to preserve these bioactive properties since, on the one hand, these berries are only available from March to May, and besides, the stability of the raw material is achieved to conserve their health benefits as food ingredients (Tabart et al., 2012; Alfaro et al., 2014); however, drying methodologies are not without detriment: the wrong drying conditions may destroy the appearance, cellular integrity, or physicochemical composition—and thus, loss of bioactive properties (Wojdyło et al., 2016). An appropriate drying method, however, can yield high-quality products with more favorable functional properties and maintain the original taste, flavor, and nutrients of berries (Zielinska et al., 2018; Sun et al., 2019). Several studies have demonstrated the effect of different drying methodologies (freeze drying, sun drying, convective drying, vacuum drying, microwave drying, and infrared drying) on the composition and abundance of phenolic components and the antioxidant capacity of many berries, including maqui berries (*Aristotelia chilensis*) (Quispe-Fuentes et al., 2020), blueberries (*Vaccinium* spp.) (Zia and Alibas, 2021), rose myrtle (*Rhodomyrtus tomentosa*) (Zhao et al., 2017), black mulberry (*Morus nigra*) (Chen et al., 2017), Sea Buckthorn (*Hippophae rhamnoides* L.) (Li et al., 2021), cranberries (*Vaccinium macrocarpon)* (Zielinska et al., 2018), or murta (*U. molinae* T.) (Puente-Díaz et al., 2013; Alfaro et al., 2014; López et al., 2019).

This research expands the literature by providing data on murta berries and how drying methods—namely, freeze drying (FD), vacuum drying (VD), infrared drying (IRD), convective drying (CD), and sun drying (SD)—may affect bioactive compounds**’** content and the resultingantioxidant potential, anti-inflammatory, and anti-tumoral activities thereof.

## 2 Materials and methods

### 2.1 Dehydration of murta samples

Murta berries were purchased from local merchants in Valdivia, Chile; in the 2016 harvest season (March–May), murta (*U. molinae* Turcz) fruits were selected for similarity (i.e., date of harvest and physical and visual characteristics) and integrity (i.e., no visible wounds or physical damage). Before processing, murta berries were refrigerated (4.5 ± 0.2*°*C) and then subjected to the dehydration methods under study (FD, VD, IRD, CD, and SD). The conditions of each method are described in detail by López et al. (2017). VD, IRD, and CD were conducted at 60°C. All samples were dried to approximately 80%–90% dry matter (d.m.) and were stored, double-bagged (polyethylene), in darkness at 5°C. Before each analysis, the samples were milled using liquid nitrogen (N_2_) using an analytical mill (IKA^®^ A-11, Wilmington, Delaware, USA) and sieved through a 500-μm sieve.

### 2.2 Proximate composition and determination of sugars

Moisture, crude proteins, lipids, crude fibers, and crude ash content were determined following AOAC methods (AOAC, 1990: Nos. 934.06, 960.52, 960.39, 962.09, and 923.03, respectively). An Aqua Lab meter (4 TE, Pullman, USA) was used to determine water activity (a_w_) at 25°C.

Following Djendoubi Mrad et al. (2012), murta fruit samples (fresh and dried by different methods) containing fructose, glucose, and sucrose content were evaluated. The milled samples (1 g) were dissolved for 30 min in 6 ml of 80% aqueous methanol, stirred at 200 rpm by agitation (Boeco orbital shaker, OS20, Hamburg, Germany), and then centrifuged for 3 min (Eppendorf, Model 5804R, Hamburg, Germany) at 2,870 *g*. Afterward, 10 µl of membrane-filtered extracts (0.45 µm) were analyzed HPLC (Flexar LC with a binary pump, autosampler, column oven, refraction index detector, PerkinElmer, Shelton, WA, USA). A 5-µm column (Supelcosil™ LC-NH_2_, 25 cm × 4.6 mm) was used to isocratically elute individual sugars (1 ml/min, 25°C, under an 82.5:17.5 acetonitrile:mobile water phase) identified after by comparison to standard retention times (the standard curve was in the range of 1.5 mg/ml to 10 mg/ml) and expressed in mg/g d.m.

### 2.3 Determination of bioactive properties

#### 2.3.1 Preparation of murta extracts

Murta fruit (10 g, fresh or dried by different methods) samples were ground and placed into a solution (aqueous ethanol 40%) of 300 ml at a solid/liquid ratio of 1:30, stirred for 24 h at 40°C (Memmert bath, WNB 22, Schwabach, Germany), centrifuged for 10 min (2,870 *g*, 10°C), filtrated (#1 filter, Whatman International Limited, Kent, England), and concentrated (Büchi R-210 rotary evaporator) at 40°C in near vacuum. The resulting extract was lyophilized and stored until analysis.

#### 2.3.2 Total phenolic content

According to Vega-Gálvez et al. (2016), Folin–Ciocalteu reagent colorimetric assay was used for the murta extracts for total phenolic content (TPC) determination. The results were expressed as the standard gallic acid equivalent per 100 *g* of dry matter (mg GAE/100 *g* d.m.).

#### 2.3.3 Phenolic compounds by high-performance liquid chromatography

The identification of phenols was performed by a high-performance liquid chromatography (HPLC) system (Santa Clara, Agilent 1200 series, CA, USA) equipped with a diode array detector (DAD). A column Kromasil 100-5C18 at 25°C was used (4.6 mm; Eka Chemical, Bohus, Sweden), and it was controlled by the software ChemStation. The chromatographic conditions were according to Rodríguez et al. (2014). The extracts were eluted at 0.7 ml/min, with 280 and 310 nm detection. The mobile phase was of 0.1% formic acid (A) and 100% acetonitrile (B)—from 87% A and 13% B, linearly graded to increase solvent B to 55% over 18 min; to 60%, from 18 to 23 min; and then a final return to initial conditions within 2 min (minutes 23 to 25). These conditions were repeated for the standard phenols (i.e., gallic, salicylic, chlorogenic, pyrogallol, protocatechuic, syringic, caffeic, vanillic, trans-sinapic, p-coumaric, ellagic, trans-ferulic, and trans-cinnamic acids, as well as pyrogallol, 3-hydroxytyrosol, catechin, tyrosol, and quercetin) and dissolved in methanol–formic acid (99:1). The concentration of each standard curve was within a range of 0.3 to 100 ppm, and the retention times and the absorption spectra to identify the phenolic compound in samples were used. The quantification was expressed as mg/100 g d.m. HPLC reagents were obtained from Merck KGaA (Darmstadt, Germany) and the standards were obtained from Sigma (St. Louis, MO, USA).

#### 2.3.4 DPPH and ORAC assays

DPPH assay was carried out according to Rodríguez et al. (2014). A 50 µM DPPH (2,2-diphenyl-1-picryl-hydrazyl) methanol solution was prepared, and 100 μl of murta extracts (in 3.9 ml of DPPH) was incubated (30 min) under darkness. Assays were measured at 517 nm, and Trolox reagent (6-hydroxy-2,5,7,8-tetramethylchroman-2-carboxylic acid) was used as a standard (0.1 to 1.0 mM standard calibration curve). Results were expressed in µmol Trolox Equivalents (TE)/g d.m.

ORAC assay was carried out according to Zhang et al. (2010) in a multiplate reader (Victor3 Multilabel Plate Reader, PerkinElmer, Turku, Finland). Extracts (40 µl) and 200 μl of fluorescein (100 µmol/L) were added in a well (96-well, polystyrene, OptiPlateTM-96 F HB, PerkinElmer, Turku, Finland) and incubated (37*°*C, 20 min). Thirty-five microliters of AAPH (0.36 mol/L) was added to start reactions. Excitation and emission wavelengths (485 nm and 535 nm) were used until fluorescence was reduced by 95% of the first reading and quantified by the difference between the sample and blank areas under the kinetic fluorescein decay curve and a calibration curve of Trolox. Results were expressed as µmol TE/g d.m. (standard calibration curve Trolox 6.25 to 200 µM).

### 2.4 Determination of biological properties

#### 2.4.1 Anti-inflammatory assays

This assay was evaluated using an *in vivo* test described by Schmeda-Hirschmann et al. (2014). Murta extracts were tested in male adult mice (20–25 g each mouse, nomenclature CF-1, ISP Chile, Instituto de Salud Pública de Chile), in adherence with published guidelines and with the University of Chile IRB and ISP approval (Code CBE-2012-16). To identify the anti-inflammatory action mechanism of murta extracts, inflammatory agents TPA (phorbol 12-myristate 13-acetate) and AA (arachidonic acid) were tested against extracts and non-steroidal anti-inflammatory drugs (indomethacin and nimesulide) as control. For each of the samples under study, the anti-inflammatory activity was evaluated in two groups: one group consisting of 8 treated mice and another group composed of 16 control mice. Briefly, mice were treated with murta extracts, controls TPA (5 mg in 20 ml acetone) or AA (2 mg in 20 ml acetone) at the same concentration. Treatments (10 µl each) were applied over the inner and outer folds of the right ear while the other ear was swabbed with acetone. Following 6 h of TPA and 1 h of AA, and then cervical dislocation, ear samples were taken (6 mm diameter) and characterized. The topical anti-inflammatory effect (E) was determined following Equation 1:


(eq. 1) %E=[(Wc-Ws)/Wc]×100

where *Wc* and *Ws* are the median weights of control and treatment ear samples, respectively; all murta extracts were evaluated at 3 mg/ear doses.

#### 2.4.2 Anti-tumoral activity

##### 2.4.2.1 Cell lines

The human NSCLC cell line NCI-H1975 and mouse hippocampus cell line HT-22 were cultured (37*°*C and 5% CO_2_, humidified incubator Thermo Scientific, MA, USA) in DMEM with 10% FBS (Dulbecco’s Modified Eagle Medium with fetal bovine serum, HyClone, Logan, UT, USA) and penicillin (50 U/ml), streptomycin (50 mg/ml), and L-glutamine (2 mM) (Nalgene, Rochester, NY, USA). The HT-22 cell line was obtained from Sigma-Aldrich, Merck, Darmstadt, Germany. The H1975 cell line was obtained from ATCC (CRL-5908), Virginia, USA.

##### 2.4.2.2 Assessments of cell viability

Seeded cancer cells (96-well plates) received applications of murta fruit extracts (0.25 mg/ml in each well) in each well using phosphate-buffered saline as substrate. After incubation (48 h), an MTT (3-[4,5-dimethylthiazol-2-yl]-2,5 diphenyl tetrazolium bromide) assay (4 h to 37*°*C, 10 μl of 5 mg/ml modified 3-[4,5-dimethylthiazol 2-yl]-2,5 diphenyltetrazolium bromide per well Sigma-Aldrich, St. Louis, MO, USA) determined mitochondrial activity. A cell lysis buffer (pH 7.4, 50% dimethylformamide, and 20% SDS) halted the reaction, and ΔA (550–650 nm) was recorded (microtiter plate reader, Metertech Σ960, Taipei, Taiwan) as a percentage of untreated cells (negative control). Each extract was evaluated in triplicate.

### 2.5 Statistical analysis

Treatment data were subjected to one-way analysis of variance (ANOVA, Statgraphics Centurion XVI, Statistical Graphics Corp., Herdon, USA) to determine significant differences (*p* < 0.05), the least significant difference test (LSD, *α* = 0.05, *p* < 0.05) for differences in average values, and the multiple range test (MRT) to group by parameter homogeneity. The non-parametric Kruskal–Wallis test showed significance (*p*) of anti-inflammatory activity, and the Mann–Whitney test, individual comparisons (*p* < 0.05).

## 3 Results and discussion

### 3.1 Physicochemical characterization of murta berries

The physicochemical characteristics of murta berries obtained after drying by the different methods (FD, VD, IRD, CD, and SD) are shown in **Table 1**. There are significant differences among dehydration methods. Both the average initial murta berry moisture of 84.25% and a_w_ (water activity) of 0.960 fell significantly (*p* < 0.05). Higher a_w_ were found in VD, IRD, and CD over SD and FD processes. The different operating modes of the dryers generated different moisture content in the samples; thus, VD, IRD, and CD contained more moisture than SD and FD samples. For example, in the case of vacuum drying, the decrease of vacuum pressure could generate bubbles inside samples creating microscopic channels, thereby allowing moisture transfer (Vega-Gálvez et al., 2022). Concerning the freeze-drying process, the moisture transfer occurs in three steps: freezing, primary drying (when ice is sublimated), and secondary drying (when the bound water begins to be removed from the dried matrix), generating a dried final product (Bhatta et al., 2020).

**Table 1:** Changes in chemical properties of murta berries after different drying processes.

Parameters	Drying methods					
	Fresh	FD	VD	IRD	CD	SD
Proximate composition (g/100 g d.m.)						
Moisture^1^	84.3 ± 0.3^a^	12.0 ± 0.2^e^	14.1 ± 0.3^d^	17.8 ± 0.1^b^	15.2 ± 0.2^c^	9.4 ± 0.1^f^
Fat	1.2 ± 0.2^d^	7.6 ± 0.1^a^	2.4 ± 0.1^c^	1.6 ± 0.3^d^	3.3 ± 0.0^b^	3.8 ± 0.3^b^
Ash	5.4 ± 0.4^a^	3.3 ± 0.2^b^	3.2 ± 0.9^b^	3.7 ± 0.3^b^	2.9 ± 0.2^b^	3.6 ± 0.3^b^
Protein	4.1 ± 0.1^b^	4.3 ± 0.1^b^	3.3 ± 0.1^d^	3.6 ± 0.2^c^	3.5 ± 0.1^c^	4.6 ± 0.1^a^
Crude fiber	15.7 ± 0.2^a^	10.6 ± 0.5^b^	8.9 ± 0.1^c^	8.6 ± 0.6^c^	8.3 ± 0.7^c^	10.8 ± 0.0^b^
**Water activity** ^2^ (a_w_)	0.960 ± 0.001^a^	0.270 ± 0.009^e^	0.655 ± 0.048^b^	0.652 ± 0.003^b^	0.565 ± 0.024^c^	0.471 ± 0.019^d^
Sugars (mg/g d.m.)						
Sucrose	146.9 ± 11.4^a^	143.8 ± 2.3^a^	26.9 ± 2.3^c^	16.0 ± 3.5^d^	44.6 ± 1.4^b^	32.6 ± 4.7^c^
Glucose	51.6 ± 5.1^c^	45.4 ± 1.7^c^	100.4 ± 4.0^a^	108.6 ± 7.2^a^	104.0 ± 0.9^a^	85.9 ± 4.4^b^
Fructose	114.9 ± 6.1^c^	107.6 ± 1.2^c^	148.5 ± 4.7^b^	160.8 ± 9.8^a^	161.9 ± 2.9^a^	142.6 ± 1.2^b^
Total	313.4	296.9	275.8	285.4	310.6	261.2

Different superscripts values (a–f) in the same row indicate significant differences (p < 0.05); FD, freeze drying; VD, vacuum drying; IRD, infrared radiation drying; CD, convective drying; SD, sun drying; ^1^Expressed as g/100 g sample; ^2^ Dimensionless.

Cellulose (crude fiber) was the main compound found in murta berries, with lesser amounts of residue ash and protein, which reflects findings in Rodríguez et al. (2014). While all drying methods led to relative decreases in crude fiber, residue ash, and protein over fresh berries, fat content significantly increased over fresh samples (**Table 1**). Notably, these berries were dried with seeds, and their lipids contributed to the quantification in the dried samples. However, it was observed that the drying procedures did not significantly affect proximate composition apart from moisture loss and concentration of total soluble solids.

Of the soluble sugars in fresh murta fruits, sucrose was found to be the most abundant (146.88 mg/100 g d.m., or approximately 2% to 3% of fresh berries), followed by fructose and glucose. Scheuermann et al. (2008) and Gironés-Vilaplana et al. (2014) also report the same trend of sugars in fresh murta berries, with the most abundant sucrose followed by fructose and glucose. Conde et al. (2007) reported that the berries in their early stages of growth accumulate a more significant amount of sucrose and that fructose and glucose increase with the maturation of the berry. However, this contradicts previous studies on other berries native to the southern cone, e.g., calafate (Ruiz et al., 2010) and maqui (Brauch et al., 2016), which showed greater glucose/fructose and less sucrose.

The dried murta samples showed an overall significant increase (*p* < 0.05) in glucose and fructose and a decrease (*p* < 0.05) in saccharose. In the FD samples, no significant differences concerning the fresh samples were observed (**Table 1**). It can be explained by solid invertase activity, catalyzing sucrose hydrolysis into glucose and fructose, and is released after cell wall disruption during the drying processes (Dewanto et al., 2002; Brauch et al., 2016). Finally, and again except FD, all methods showed a slight but significant decrease in total soluble sugars over fresh murta berries—perhaps related to thermal degradation during the Maillard reaction (Woo et al., 2015) due to sugar participation in browning reactions during prolonged heating (Arocho et al., 2012).

### 3.2 Bioactive properties

#### 3.2.1 Identification of phenols

Nine compounds (pyrogallol, vanillic, catechin, 3-hydroxytyrosol, gallic, protocatechuic, tyrosol, quercetin, and trans-cinnamic acids) were detected in the phenolic fractions of the fresh and dried murta fruits (**Table 2**). Other authors have also identified the same compounds, showing them to be characteristic of murta berries (Alfaro et al., 2014; Augusto et al., 2014; Junqueira-Gonçalves et al., 2015; Jofré et al., 2016; Rodríguez et al., 2016; Fredes et al., 2020; Gómez-Pérez et al., 2022). Trans-cinnamic acid was least present in all drying methods and not quantifiable in fresh murta fruit. Similarly, protocatechuic acid, vanillic acid, and quercetin were undetectable in fresh murta. For FD products, protocatechuic and vanillic acids were not detected. In all the drying methods, there was a significant decrease (*p* < 0.05) of 3-hydroxytyrosol, catechin, and tyrosol compared to the fresh sample, and higher values of dried samples of pyrogallol, quercetin, gallic, and vanillic acids are observed compared to the fresh sample. Notably, vanillic acid was not measured in FD samples.

**Table 2:** Profile of phenolic acids in fresh and dried murta fruits.

Phenolic compounds (mg/100 g d.m.)*	Drying methods					
	Fresh	FD	CD	VD	SD	IRD
Gallic acid	4.6 ± 0.2^d^	5.5 ± 0.4^d^	10.1 ± 0.8^b^	10.1 ± 0.6^b^	9.3 ± 0.4^c^	13.4 ± 0.3^a^
Pyrogallol	69.4 ± 2.6^e^	231.0 ± 4.0^d^	506.9 ± 17.5^a^	262.9 ± 6.6^c^	219.7 ± 6.2^d^	315.9 ± 5.3^b^
3-Hydroxytyrosol	24.7 ± 0.4^a^	17.6 ± 0.2^c^	14.4 ± 0.1^e^	15.8 ± 0.2^d^	13.4 ± 0.2^e^	19.2 ± 1.1^b^
Protocatechuic acid	NQ	NQ	6.5 ± 0.0^ab^	7.3 ± 0.0^a^	6.1 ± 0.8^b^	6.6 ± 0.0^ab^
Catechin	172.7 ± 1.5^a^	97.7 ± 3.1^c^	98.4 ± 1.0^c^	86.5 ± 1.3^d^	44.6 ± 1.6^e^	105.4 ± 1.3^b^
Tyrosol	52.5 ± 3.7^a^	39.2 ± 0.2^c^	46.7 ± 3.5^b^	29.5 ± 0.7^d^	27.0 ± 1.8^d^	40.9 ± 3.3^bc^
Vanillic acid	NQ	NQ	10.4 ± 0.2^a^	5.5 ± 0.2^c^	4.8 ± 0.5^c^	8.0 ± 0.4^b^
Quercetin	NQ	0.7 ± 0.1^c^	1.2 ± 0.0^b^	1.6 ± 0.1^a^	0.8 ± 0.1^c^	1.2 ± 0.1^b^
Trans-cinnamic acid	NQ	0.1 ± 0.0^e^	0.3 ± 0.1^c^	0.5 ± 0.0^b^	0.6 ± 0.0^a^	0.3 ± 0.0^d^

Different superscript values (a–e) in the same row indicate significant differences (p < 0.05); FD, freeze drying; VD, vacuum drying; IRD, infrared radiation drying; CD, convective drying; SD, sun drying, NQ, not quantifiable. *Sample preparation and HPLC determination were performed in triplicate.

The most present phenol in fresh murta berries was catechin (172.72 mg/100 g d.m.), a flavanol (Grzesik et al., 2018). In contrast, the lowest were gallic acid (4.57 mg/100 g d.m.) and 3-Hydroxytyrosol (24.73 mg/100 g d.m.). In all dehydrated samples, pyrogallol was the most present phenolic compound, most significant in the CD drying method (506.89 mg/100 g d.m.), over 7.3 times more than fresh murta berries. The second phenol most common in all dehydrated samples was catechin, which, although significantly decreased compared to fresh berries, suggests resistance to thermal degradation, as mentioned in López et al. (2019). Of the phenolic compounds found in lesser concentrations, pyrogallol—falling into the categories of gallocatechin (Iglesias et al., 2013), a flavonoid (flavan-3-ol) (Du et al., 2012), and trihydroxybenzene (Florence et al., 2018)—has shown to have antibacterial, antioxidant, and anti-inflammatory activity (Furuno et al., 2002; Nicolis et al., 2008; Lima et al., 2016).

Then, drying methods alter the phenolic profiles of murta berries. It may also be presumed that the changes occurring during the distinct drying and temperature processes may be associated with compound instability. Indeed, Ortiz et al. (2021) suggested that processes above 60*°*C may induce oxidization in more susceptible compounds.

Speisky et al. (2022) mentioned the effects that the oxidative and non-oxidative modification of the phenolic groups of flavonoids might have on the ability of the resulting metabolites to promote direct or indirect antioxidant reactions and include other molecules with reducing capacity. Moreover, according to Fabani et al. (2017), the content of individual phenolic compounds increases with the drying process, mainly due to the concentration effect and the hydrolysis of polymerized phenolic compounds. Consequently, phenol content values vary widely, either fresh or dried fruits.

#### 3.2.2 Total phenolic content

**Figure 1:**
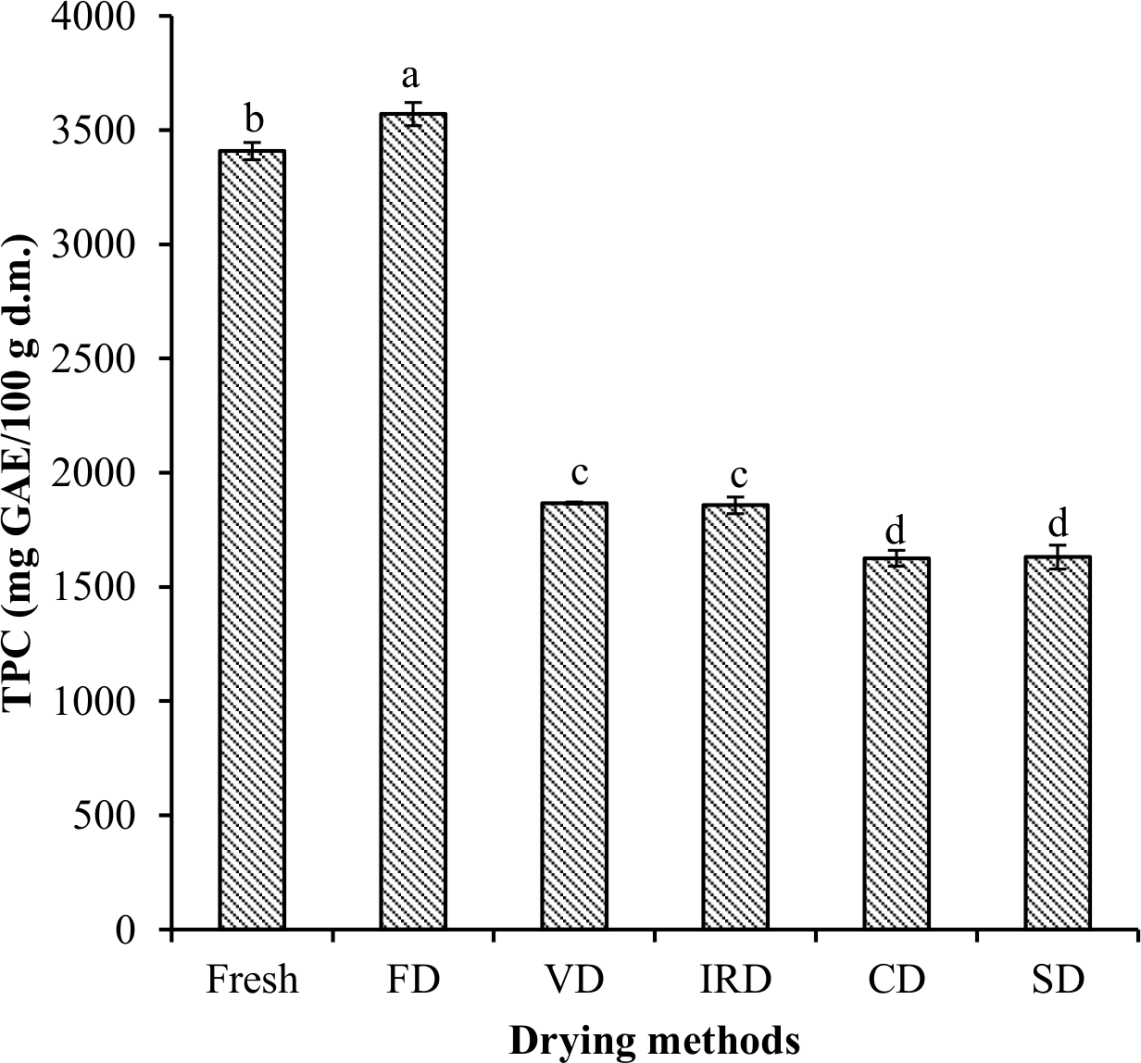
Total phenolic contents of murta berries after different drying processes. Different letters on same-shaded bars indicate significant differences (*p* < 0.05). FD, freeze drying; VD, vacuum drying; IRD, infrared radiation drying; CD, convective drying; SD, sun drying.

**Figure 1** shows the results of TPC for fresh and dehydrated murta by different drying methods. Fresh murta contained 3,327 mg GAE/100 g d.m. of phenols, much higher than in other murta berry studies: López et al. (2019) reported 1,068.59 mg GAE/100 g d.m.; Augusto et al. (2014), 1,935 mg GAE/100 g d.m.; and Reyes et al. (2011), 1,460 mg GAE/100 g d.m. Additionally, Ramirez et al. (2015) reported 924 mg GAE/100 g d.m. in myrtle berry (*Myrtus communis*). The differences observed can be attributed to the different ecotypes of murta, raw material management, the location where they grow, harvest time, and soil conditions, among others, whether they are intrinsic or extrinsic conditions of the raw material (López et al., 2018; Fredes et al., 2020).

The TPC in dehydrated murta showed significant differences (*p* < 0.05) across all drying methods over fresh murta, ranging from 1,579 to 3,570 mg GAE/100 g d.m. for SD and FD, respectively. SD decreased 52% over fresh murta berries. Moreover, freeze-dried berries retained better phenol content than other drying methods. There was no significant difference between VD and IRD or CD and SD (*p* > 0.05).

These decreases in results may be due to oxidation, degradation, enzymatic inactivation, or volatilization during dehydration processes (Henríquez et al., 2014), especially since phenolic compounds are generally thermolabile (Fredes et al., 2020). Indeed, previous studies (Pap et al., 2021) have reported additional effects from activated polyphenol oxidase (PPO) on phenol structure.

#### 3.2.3 Antioxidant potential

**Figure 2:**
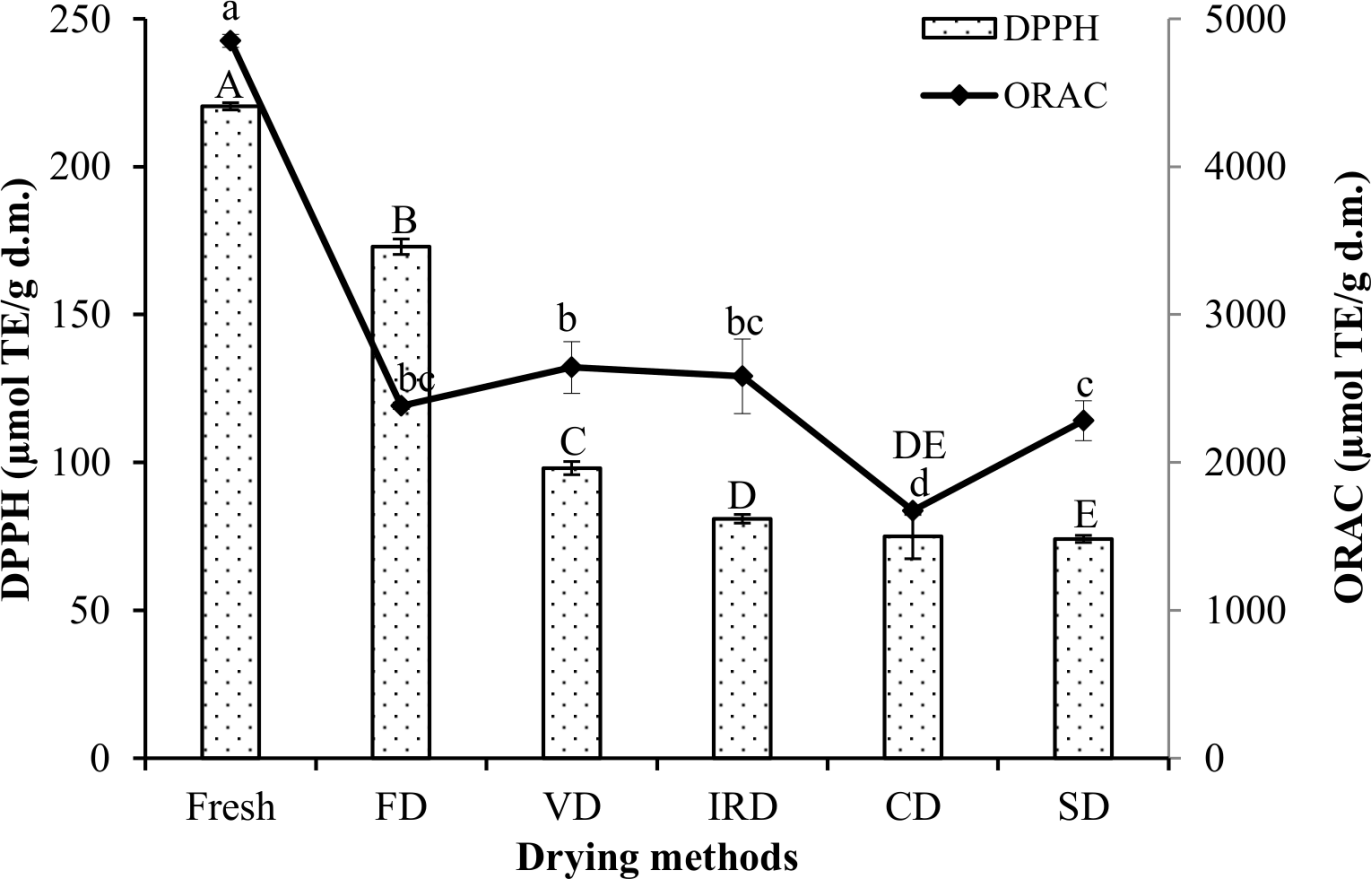
Antioxidant potential as measured by DPPH and ORAC assays in murta berries after different drying processes. Different letters on the same bars (uppercase) and lines indicate (lowercase) significant differences (*p* < 0.05). FD, freeze drying; VD, vacuum drying; IRD, infrared radiation drying; CD, convective drying; SD, sun drying.

The antioxidant potential of phenolic fractions of the fresh and dried murta fruits was determined by DPPH and ORAC assays in **Figure 2**. In the fresh murta berries, the DPPH assay yielded 220 µmol TE/g d.m. and the ORAC assay yielded 4,851 µmol TE/g d.m. (**Figure 2**). While ORAC values differ from previously reported values—perhaps related to the extraction method—the fresh murta DPPH assay is consistent with previous reports (Alfaro et al., 2013; Alfaro et al., 2014; Augusto et al., 2014; Rodríguez et al., 2014), yielding 21 to 457 µmol TE/g d.m. for different varieties of murta fruits.

All drying methods led to significant losses in the antioxidant potential, almost certainly due to enzymatic or thermal damage or inhibition of phytochemicals (Gao & Mazza, 1994). Notwithstanding, FD murta berries retained 78% of the total antioxidant potential by DPPH of the fresh berries, the highest among drying methods. These ranged between 33% and 44%, similar to previous reports (Alfaro et al., 2014).

In the ORAC assay, VD, IRD, and FD samples showed no significant differences; moreover, all samples exhibited significant decreases over fresh murta, between 45% and 65%, encompassing similar ranges reported by López et al. (2019).

In drying methods, temperature and time are principal contributing factors to the loss of antioxidant potential.

CD drying samples showed the lowest total antioxidant potential in both assays, likely due to the relatively intense and prolonged exposure to oxygen and subsequent enzymatic degradation of some of the phytochemicals. Despite losses in all methods, ORAC suggests relatively high antioxidant activity. This result may be due to pyrogallol content (CD, VD, SD, and IRD) (**Table 2**), which has previously (López-Alarcón and Lissi, 2005) shown to react highly with peroxyl radicals.

However, for quantitative antioxidant analysis of natural materials, these assays should be replaced with new antioxidant assays, including lipid oxidation assays, with clearly identifiable reaction mechanisms, thoroughly tested reaction conditions, and kinetic and stoichiometric analyses (Schaich et al., 2015).

### 3.3 Biological properties

#### 3.3.1 Anti-inflammation evaluations

All murta fruit extracts obtained from the different drying methods exhibited inhibition of topical inflammation induced by TPA and AA (phorbol 12-myristate 13-acetate and arachidonic acid, respectively). Typical anti-inflammatory agents such as indomethacin (IND) and nimesulide (NIM) were used as controls to treat TPA- and AA-induced inflammation, showing 92.9% and 48.8% reductions, respectively (**Table 3**). The effect of fresh murta fruit extracts obtained against TPA- and AA-induced inflammation was similar (18% and 15% edema reduction, respectively). FD murta sample extracts showed the highest anti-inflammatory response in both models of inflammation (65% and 41.3% edema reduction in the TPA and AA models, respectively), followed by VD extracts (63.2% and 34.2%, respectively). The IRD and CD methods showed a significant effect (51.2% and 47.5%, respectively) against TPA-induced inflammation. No significant topical anti-inflammatory effect was induced by IRD, CD, and SD murta samples’ extracts on AA-inflamed tissues. Previous reports (Delporte et al., 2007; Goity et al., 2013) show strong correlations between bioactive compounds in plant matter and inhibition of topical inflammation from plant extracts. Thus, the potent inhibition responses against TPA (>60% of FD and VD murta extracts over fresh fruit extracts) and AA (FD murta extracts) not insignificantly of the same order as the reference drug (NIM) are almost certainly caused by the presence of highly concentrated phenolic acids and flavonoids after FD and VD processes (**Table 3**).

**Table 3:** Topical anti-inflammatory effects of murta fruit extracts against TPA- and AA-induced inflammation of mice ear edema.

Drying methods (murta fruit extracts)	Topical anti-inflammatory effects		
	Dose (mg/ear)	%EA_TPA_ ± SEM	%EA_AA_ ± SEM
Fresh	3	18.0 ± 4.7^*,d^	15.0 ± 4.5^*,b^
FD	3	65.0 ± 4.1^*,b^	41.3 ± 3.7^*,a^
VD	3	63.2 ± 3.0^*,b^	34.2 ± 4.2^*,a^
IRD	3	51.2 ± 3.0 ^c^	18.8 ± 6.1^*,b^
CD	3	47.5 ± 4.8^*,c^	14.6 ± 3.9^*,b^
SD	3	22.8 ± 4.8^*,d^	16.7 ± 4.0^*,b^
IND	0.5	↑92.9 ± 10.2^*,a^	n.d
NIM	1	n.d	↑48.8 ± 4.0^*,a^

Different superscript values (a–d) in the same column indicate significant differences (p < 0.05); ^*^(p < 0.05) with respect to control. ↑maximum effect; FD, freeze drying; VD, vacuum drying; IRD, infrared radiation drying; CD, convective drying; SD, sun drying; EA_TPA_, topical anti-inflammatory effect against TPA; EA_AA_, topical anti-inflammatory effect against AA; IND, indomethacin; NIM, nimesulide; n.d, not determined.

Furthermore, previous research (Inoue et al., 1988; Griswold et al., 1991) suggests that phenolic compounds may act on free radicals and inhibit some inflammation events, and, indeed, phenolic compounds identified in the murta extracts, quercetin, and gallic acid are known to elicit an anti-inflammatory response. Quercetin inhibits interleukin-1β (IL-1β), IL-6, and TNF-α, as well as STAT-1 and NF-κB (Hämäläinen et al., 2007). Next, two mechanisms of action have been proposed for inhibiting inflammation by gallic acid: neutralization of Dioxide(1−) radicals and suppression of MPO, and mediation in active NADPH-oxidase (Kroes et al., 1992).

The literature has an ample discussion on other bioactive compounds extracted from berries and their beneficial effects. Thus, Folmer et al. (2014) mentioned in their review the health benefits of bioactive compounds (flavonoids, ellagitannins, phenolic acids, and stilbenoids) from various types of berries (strawberries, red raspberries, black raspberries, blackberries, mulberries, and blueberries) concerning their anti-inflammatory properties and anti-carcinogenic effects. Similarly, Martin et al. (2014) found that polyphenols present in extracts of Aronia inhibited mechanisms leading to rheumatological and broad inflammation disorders in the neurological, joint, and digestive systems.

Arancibia-Radich et al. (2016) studied the effects of murta leaves across several variants and found associations between the phenolic compounds and triterpenoids present and the anti-inflammatory activity.

#### 3.3.2 Anti-tumoral activity

Cytotoxic activity on highly proliferative cell lines of the prepared extracts of murta berries was determined following standard procedure (Adenan et al., 2018) by subjecting human and mouse cell lines to MTT assays, which measure cellular metabolic activity and estimate cell survival.

**Figure 3:**
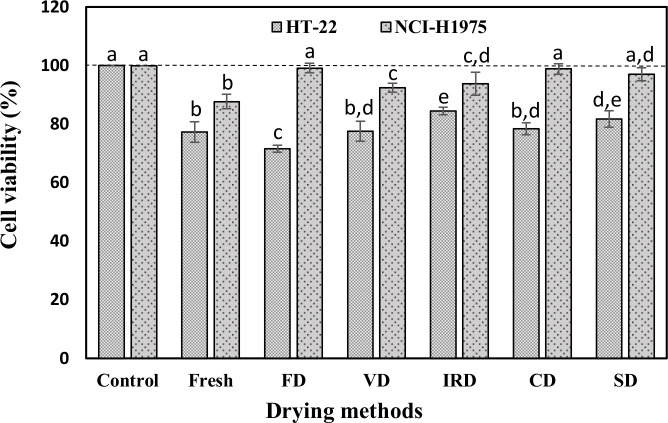
Anti-tumoral effect of murta fruit extracts (0.25 mg/ml) on HT-22 and NCI-H1975 cell viability after 48 h of incubation. All determinations are expressed as a percentage of the control (untreated cells). Different letters on same-shaded bars indicate significant differences (*p* < 0.05). FD, freeze drying; VD, vacuum drying; IRD, infrared radiation drying; CD, convective drying; SD, sun drying.

Human cancer line NCI-H1975 and mouse line HT-22 cells were significantly repressed after 48 h of murta extract at a 0.25 mg/ml concentration. **Figure 3** compares the dehydration method, controls, and measured cell viability.

Our data showed that no extract tested reduced cell viability below 71% of control in the two treated cell lines. Analyzing murta fruit extracts on the NCI-H1975 cell line, the fresh extract decreased cell viability by approximately 13% compared to the control. Comparing different drying methods, the VD and IRD are the most efficient methods to maintain the anti-tumoral effect on this type of cancer cells. This effect may be due to the phenolic components of murta extracts, especially given their potential oxidative capacity in the presence of specific metal ions (Caprioli et al., 2016). In the case of HT-22, an immortalized cell line with a high proliferation rate, the FD murta samples demonstrated the most significant activity, with 71.56% of cell survival, followed by fresh (77.34%), VD samples (77.59%), and CD (78.41%). These results suggest that this immortalized cell line is more susceptible to decreased cell survival in the presence of murta samples.

Chemical characterization of murta extracts showed the presence of polyphenols, with catechin as the most abundant (**Table 2**). Catechin has been reported to have anticancer effects across many mammalian systems, e.g., digestive components, the endocrine system, circulatory and respiratory systems, and many others (Sheng, 2020). Bragança De Moraes et al. (2012) reported that catechin inhibited the proliferation of GRX liver cells, attributing the action to the inhibition of inflammation and the cell cycle. Additionally, Abd-Rabou et al. (2016) found anti-carcinogenic ellagic and gallic acids to be cytotoxic mediators against HepG2 and HCT 116 cell lines and decreasing viable human colon tumor cells; Flis et al. (2012) showed the effectiveness of antioxidant murta berry extracts—correlated with its phenols—against colon cancer cell lines; Avello et al. (2020) found that total aqueous murta leaf extract had synergistic effects in inhibiting cell viability against gastric adenocarcinoma cells (AGS) and that the metabolites of some elements even inhibited the viability of Caco-2 cells.

## 4 Conclusions

The present study demonstrated the effects of different methods of drying murta berries on chemical properties, phenolic compounds, and some biological properties. The freeze-drying process retained the extractability of phenolic compounds and showed the highest antioxidant, anti-inflammatory, and anti-tumoral activities due to the individual phenolic compounds such as catechin, pyrogallol, tyrosol, and gallic acid that were released and conserved from the fruit matrix.

Nevertheless, freeze drying is too cost-intensive. Vacuum and infrared drying were also shown to produce high-quality dried murta berries and thus represent excellent alternatives. These results would help to select the drying process of murta berries to produce the desired bioactive compounds since the murta is a promising antioxidant, anti-inflammatory, and anti-tumoral compound source. However, in future research for quantitative antioxidant analysis, these assays should be replaced with new antioxidant assays.

## Data availability statement

The original contributions presented in the study are included in the article/supplementary material. Further inquiries can be directed to the corresponding author.

## Ethics statement

The animal study was reviewed and approved by Animal Care and Use Committee of the Facultad de Ciencias Químicas y Farmacéuticas of Universidad de Chile and Instituto de Salud Pública de Chile (Code of approval CBE-2012-190 16).

## Author contributions

JL: Wrote the paper, collected the data, contributed data. AV-G: Conceived and designed the analysis. KA-H: Wrote the paper, performed the statistical analysis AR: Wrote the paper, contributed analysis tools. IQ-F: analyzed the data of the HPLC analysis and wrote the results. CD: Contributed data in the analysis of anti-inflammatory activity. GV-B: Contributed data in the analysis of anti-inflammatory activity. YA: Contributed data in the analysis of antiproliferative activity. AZ: contributed to data analysis, contributed data in the analysis of antiproliferative activity. All authors contributed to the article and approved the submitted version.

## References

[B1] Abd-Rabou A. A. Shalby A. B. Ahmed H. H. (2016). Anti-cancer activity of quercetin, gallic acid, and ellagic acid against HEPG2 and HCT 116 cell lines: *In vitro.* int. J. Pharma. Bio. Sci. 7, 584–592. doi: 10.22376/ijpbs.2016.7.4.b584-592

[B2] Adenan M. N. H. Adam Z. Khamis S. Hassali H. A. Saaya F. M. (2018). Antiproliferative study of *Brucea javanica* extracts against head and neck cancer cells. J. Inf. Syst. Technol. Manage. 3, 55–66.

[B3] Alfaro S. Mutis A. Palma R. Quiroz A. Seguel I. Scheuermann E. (2013). Influence of genotype and harvest year on polyphenol content and antioxidant activity in murtilla (*Ugni molinae* turcz) fruit. J. Soil Sci. Plant Nutr. 13, 67–78. doi: 10.4067/S0718-95162013005000007

[B4] Alfaro S. Mutis A. Quiroz A. Seguel I. Scheuermann E. (2014). Effects of drying techniques on murtilla fruit polyphenols and antioxidant activity. J. Food Res. 3, 73–82. doi: 10.5539/jfr.v3n5p73

[B5] AOAC (1990). Official method of analysis. 15th ed (Arlington, MA: Association of Official Analytical Chemists).

[B6] Arancibia-Radich J. Peña-Cerda M. Jara D. Valenzuela-Bustamante P. Goity L. Valenzuela-Barra G.. (2016). Comparative study of anti-inflammatory activity and qualitative-quantitative composition of triterpenoids from ten genotypes of *Ugni molinae.* bol. Latinoam. y del Caribe Plantas Med. y Aromat. 15, 274–287.

[B7] Arocho Y. D. Bellmer D. Maness N. Mcglynn W. Rayas-Duarte P. (2012). Watermelon pomace composition and the effect of drying and storage on lycopene content and color. J. Food Qual. 35, 331–340. doi: 10.1111/j.1745-4557.2012.00455.x

[B8] Augusto T. R. Scheuermann Salinas E. S. Alencar S. M. D’Arce M. A. B. R. De Camargo A. C. Vieira T. M. F.. (2014). Phenolic compounds and antioxidant activity of hydroalcoholic extractsof wild and cultivated murtilla (*Ugni molinae* turcz.). Food Sci. Technol. 34, 667–673. doi: 10.1590/1678-457X.6393

[B9] Avello M. Pastene E. Eillen T. (2020). Identification of water-soluble compounds contained in aqueous extracts and fractions obtained from leaves of ugni molinae to determine their effect on the viability of human gastric cancer cells. J. Chil. Chem. Soc 65, 4849–4852. doi: 10.4067/S0717-97072020000204849

[B10] Bhatta S. Stevanovic Janezic T. Ratti C. (2020). Freeze-drying of plant-based foods. Foods 9 (1), 87. doi: 10.3390/foods9010087 31941082 PMC7022747

[B11] Bouyahya A. Omari N. E. EL Hachlafi N. Jemly M. E. Hakkour M. Balahbib A.. (2022). Chemical compounds of berry-derived polyphenols and their effects on gut microbiota, inflammation, and cancer. Molecules 27, 3286. doi: 10.3390/molecules27103286 35630763 PMC9146061

[B12] Bragança De Moraes C. M. da Silva D. A. Vianna R. C. Bitencourt S. Mesquita F. C. dos Santos de Oliveira F.. (2012). Antiproliferative effect of catechin in GRX cells. Biochem. Cell Biol. 90, 575–584. doi: 10.1139/o2012-010 22574829

[B13] Brauch J. E. Buchweitz M. Schweiggert R. M. Carle R. (2016). Detailed analyses of fresh and dried maqui (*Aristotelia chilensis* (Mol.) stuntz) berries and juice. Food Chem. 190, 308–316. doi: 10.1016/j.foodchem.2015.05.097 26212975

[B14] Caprioli G. Alunno A. Beghelli D. Bianco A. Bramucci M. Frezza C.. (2016). Polar constituents and biological activity of the berry-like fruits from *Hypericum androsaemum* l. Front. Plant Sci. 7, 1–12. doi: 10.3389/fpls.2016.00232 26973675 PMC4771922

[B15] Castro R. I. Ramos P. Parra-Palma C. Morales-Quintana L. (2021). Ugni molinae fruit as a source of bioacitve compounds with good quality traits. BioMed. Res., 11. Int., Article ID 6683877. doi: 10.1155/2021/6683877 PMC8088357 33981771

[B16] Chen Q. Li Z. Bi J. Zhou L. Yi J. Wu X. (2017). Effect of hybrid drying methods on physicochemical, nutritional and antioxidant properties of dried black mulberry. LWT - Food Sci. Technol. 80, 178–184. doi: 10.1016/j.lwt.2017.02.017

[B17] Conde C. Silva P. Fontes N. Dias A. C. P. Tavares R. M. Sousa M. J.. (2007). Biochemical changes throughout grape berry development and fruit and wine quality. Food 1, 1–22.

[B18] Delporte C. Backhouse N. Inostroza V. Aguirre M. C. Peredo N. Silva X.. (2007). Analgesic activity of *Ugni molinae* (murtilla) in mice models of acute pain. J. Ethnopharmacol. 112, 162–165. doi: 10.1016/j.jep.2007.02.018 17403589

[B19] Dewanto V. Wu X. Liu R. H. (2002). Processed sweet corn has higher antioxidant activity. J. Agric. Food Chem. 50, 4959–4964. doi: 10.1021/jf0255937 12166989

[B20] Djendoubi Mrad N. Bonazzi C. Boudhrioua N. Kechaou N. Courtois F. (2012). Influence of sugar composition on water sorption isotherms and on glass transition in apricots. J. Food Eng. 111, 403–411. doi: 10.1016/j.jfoodeng.2012.02.001

[B21] Du H. Wang H. Yu J. Liang C. Ye W. Li P. (2012). Enrichment and purification of total flavonoid c-glycosides from abrus mollis extracts with macroporous resins. Ind. Eng. Chem. Res. 51, 7349–7354. doi: 10.1021/ie3004094

[B22] Fabani M. P. Baroni M. V. Luna L. Lingua M. S. Monferran M. V. Paños H.. (2017). Changes in the phenolic profile of argentinean fresh grapes during production of sun-dried raisins. J. Food Compos. Anal. 58, 23–32. doi: 10.1016/j.jfca.2017.01.006

[B23] Flis S. Jastrzebski Z. Namiesnik J. Arancibia-Avila P. Toledo F. Leontowicz H.. (2012). Evaluation of inhibition of cancer cell proliferation *in vitro* with different berries and correlation with their antioxidant levels by advanced analytical methods. J. Pharm. Biomed. Anal. 62, 68–78. doi: 10.1016/j.jpba.2012.01.005 22300907

[B24] Florence I. Hery S. Akhmad D. (2018). Antibacterial and antioxidant activities of pyrogallol and synthetic pyrogallol dimer. Res. J. Chem. Environ. 22, 39–47.

[B25] Folmer F. Basavaraju U. Jaspars M. Hold G. El-Omar E. Dicato M.. (2014). Anticancer effects of bioactive berry compounds. Phytochem. Rev. 13, 295–322. doi: 10.1007/s11101-013-9319-z

[B26] Fredes C. Parada A. Salinas J. Robert P. (2020). Phytochemicals and traditional use of two southernmost Chilean berry Fruits : murta (*Ugni molinae* turcz) and calafate (*Berberis buxifolia* lam.) Carolina. Foods 9, 1–16. doi: 10.3390/foods9010054 PMC7023186 31935880

[B27] Furuno K. Akasako T. Sugihara N. (2002). The contribution of the pyrogallol moiety to the superoxide radical scavenging activity of flavonoids. Biol. Pharm. Bull. 25, 19–23. doi: 10.1248/bpb.25.19 11824550

[B28] Gao L. Mazza G. (1994). Quantitation and distribution of simple and acylated anthocyanins and other phenolics in blueberries. J. Food Sci. 59, 1057–1059. doi: 10.1111/j.1365-2621.1994.tb08189.x

[B29] Gironés-Vilaplana A. Baenas N. Villaño D. Moreno D. (2014). Iberian-American Fruits rich in bioactive phytochemicals for nutrition and health. (Alicante Spain: LIMENCOP S.L.).

[B30] Goity L. E. Queupil M. J. Jara D. Alegría S. E. Peña M. Barriga A.. (2013). An HPLC-UV and HPLC-ESI-MS based method for identification of anti-inflammatory triterpenoids from the extracts of ugni molinae. Bol. Latinoam. y del Caribe Plantas Med. y Aromat. 12, 108–116.

[B31] Golovinskaia O. Wang C.-K. (2021). Review of functional and pharmacological activities of berries. Molecules 26, 3904. doi: 10.3390/molecules26133904 34202412 PMC8271923

[B32] Gómez-Pérez L. S. Moraga N. Ah-Hen K. S. Rodríguez A. Vega-Gálvez A. (2022). Dietary fibre in processed murta (*Ugni molinae* turcz) berries: bioactive components and antioxidant capacity. J. Food Sci. Technol. 59, 3093–3101. doi: 10.1007/s13197-022-05416-1 35872745 PMC9304509

[B33] Griswold D. Marshall P. Lee J. Webb E. Hillegass L. M. Wartell J.. (1991). Pharmacology of the pyrroloimidazole, SK&F 105809– II: Antiinflammatory activity and inhibition of mediator production in vivo. Biochem. Pharmacol. 42 (4), 825–831. doi: 10.1016/0006-2952(91)90042-4 1907825

[B34] Grzesik M. Naparło K. Bartosz G. Sadowska-Bartosz I. (2018). Antioxidant properties of catechins: comparison with other antioxidants. Food Chem. 241, 480–492. doi: 10.1016/j.foodchem.2017.08.117 28958556

[B35] Hämäläinen M. Nieminen R. Vuorela P. Heinonen M. Moilanen E. (2007). Anti-inflammatory effects of flavonoids: genistein, kaempferol, quercetin, and daidzein inhibit STAT-1 and NF-κB activations, whereas flavone, isorhamnetin, naringenin, and pelargonidin inhibit only NF-κB activation along with their inhibitory effect on i. Mediators Inflamm. 2007, 45673. doi: 10.1155/2007/45673 18274639 PMC2220047

[B36] Henríquez C. Córdova A. Almonacid S. Saavedra J. (2014). Kinetic modeling of phenolic compound degradation during drum-drying of apple peel by-products. J. Food Eng. 143, 146–153. doi: 10.1016/j.jfoodeng.2014.06.037

[B37] Iglesias J. Medina I. Pazos M. (2013). Galloylation and polymerization: role of structure to antioxidant activity of polyphenols in lipid systems. Polyphenols Hum. Heal. Dis 1, 323–338. doi: 10.1016/B978-0-12-398456-2.00025-6

[B38] Inoue H. Mori T. Koshihara Y. (1988). Sulfidopeptide-leukotrienes are major mediators of arachidonic acid-induced mouse ear edema. Prostaglandins 36, 731–9. doi: 10.1016/0090-6980(88)90016-0 3148965

[B39] Jofré I. Pezoa C. Cuevas M. Scheuermann E. Freires I. A. Rosalen P. L.. (2016). Antioxidant and vasodilator activity of ugni molinae turcz. (Murtilla) and its modulatory mechanism in hypotensive response. Oxid. Med. Cell. Longev. 2016, 6513416. doi: 10.1155/2016/6513416 27688827 PMC5027056

[B40] Junqueira-Gonçalves M. P. Yáñez L. Morales C. Navarro M. Contreras R. A. Zúñiga G. E. (2015). Isolation and characterization of phenolic compounds and anthocyanins from murta (*Ugni molinae* turcz.) fruits. Assess. antioxidant antibacterial activity. Molecules 20, 5698–5713. doi: 10.3390/molecules20045698 25838172 PMC6272493

[B41] Kroes B. H. Van Den Berg A. J. J. Quarles Van Ufford H. C. Van Dijk H. Labadie R. P. (1992). Anti-inflammatory activity of gallic acid. Planta Med. 58, 499–504. doi: 10.1055/s-2006-961535 1336604

[B42] Li Y. Li P. Yang K. He Q. Wang Y. Sun Y.. (2021). Impact of drying methods on phenolic components and antioxidant activity of Sea buckthorn (*Hippophae rhamnoides* l.) berries from different varieties in China. Molecules 26, 7189. doi: 10.3390/molecules26237189 34885771 PMC8659002

[B43] Lima V. N. Oliveira-Tintino C. D. M. Santos E. S. Morais L. P. Tintino S. R. Freitas T. S.. (2016). Antimicrobial and enhancement of the antibiotic activity by phenolic compounds: Gallic acid, caffeic acid and pyrogallol. Microb. Pathog. 99, 56–61. doi: 10.1016/j.micpath.2016.08.004 27497894

[B44] López J. Vega-Gálvez A. Bilbao-Sainz C. Uribe E. Chiou B.-S. Quispe-Puentes I. (2017). Influence of vacuum drying temperature on : physico-chemical composition and antioxidant properties of murta berries. Food Process Eng. 40(6), 1–9. doi: 10.1111/jfpe.12569

[B45] López J. Vega-Gálvez A. Rodríguez A. Stucken K. Barraza C. Aguilera L. E. (2019). Relationship between antimicrobial activity, phenolic profile and antioxidant capacity of murta (*Ugni molinae* turcz) extracts prepared by different drying methods. J. Berry Res. 9, 587–601. doi: 10.3233/JBR-190403

[B46] López J. Vega-Gálvez A. Rodríguez A. Uribe E. Bilbao-Sainz C. (2018). Murta (Ugni molinae turcz.): a review on chemical composition, functional components and biological activities of leaves and fruits. chil. J. Agric. Anim. Sci. 34 (1), 43–56. doi: 10.4067/S0719-38902018005000205

[B47] López-Alarcón C. Lissi E. (2005). Interaction of pyrogallol red with peroxyl radicals. a basis for a simple methodology for the evaluation of antioxidant capabilities. Free Radic. Res. 39, 729–736. doi: 10.1080/10715760500143452 16036352

[B48] Martin D. A. Taheri R. Brand M. H. Draghi A. Sylvester F. A. Bolling B. W. (2014). Anti-inflammatory activity of aronia berry extracts in murine splenocytes. J. Funct. Foods 8, 68–75. doi: 10.1016/j.jff.2014.03.004

[B49] Mazzoni L. Giampieri F. Alvarez Suarez J. M. Gasparrini M. Mezzetti B. Forbes Hernandez T. Y.. (2019). Isolation of strawberry anthocyanin-rich fractions and their mechanisms of action against murine breast cancer cell lines. Food Funct. 10, 7103–7120. doi: 10.1039/C9FO01721F 31621765

[B50] Miladinovic B. Faria M.Â. Ribeiro M. Sobral M. M. C. Ferreira I.M.P.L.V.O. (2023). Delphinidin-3-rutinoside from blackcurrant berries (*Ribes nigrum*): *In vitro* antiproliferative activity and interactions with other phenolic compounds. Molecules 28, 1286. doi: 10.3390/molecules28031286 36770953 PMC9920764

[B51] Nicolis E. Lampronti I. Dechecchi M. C. Borgatti M. Tamanini A. Bianchi N.. (2008). Pyrogallol, an active compound from the medicinal plant emblica officinalis, regulates the expression of pro-inflammatory genes in bronchial epithelial cells. Int. Immunopharmacol. 8, 1672–1680. doi: 10.1016/j.intimp.2008.08.001 18760383

[B52] Ortiz T. Argüelles-Arias F. Begines B. García-Montes J. M. Pereira A. Victoriano M.. (2021). Native chilean berries preservation and *in vitro* studies of a polyphenol highly antioxidant extract from maqui as a potential agent against inflammatory diseases. Antioxidants 10, 843. doi: 10.3390/antiox10060843 34070392 PMC8226669

[B53] Otero C. Klagges C. Morales B. Sotomayor P. Escobar J. Fuentes J. A.. (2023). Anti-inflammatory Chilean endemic. Plants. Pharmaceutics 15, 897. doi: 10.3390/pharmaceutics15030897 36986757 PMC10051824

[B54] Pap N. Fidelis M. Azevedo L. do Carmo M. A. V. Wang D. Mocan A.. (2021). Berry polyphenols and human health: evidence of antioxidant, anti-inflammatory, microbiota modulation, and cell-protecting effects. Curr. Opin. Food Sci. 42, 167–186. doi: 10.1016/j.cofs.2021.06.003

[B55] Puente-Díaz L. Ah-Hen K. Vega-Gálvez A. Lemus-Mondaca R. Di Scala K. (2013). Combined infrared-convective drying of murta (*Ugni molinae* turcz) berries: kinetic modeling and quality assessment. Dry. Technol. 31, 329–338. doi: 10.1080/07373937.2012.736113

[B56] Quispe-Fuentes I. Vega-Gálvez A. Aranda M. Poblete J. Pastén A. Bilbao-Sainz C.. (2020). Effects of drying processes on composition, microstructure and health aspects from maqui berries. J. Food Sci. Technol. 57 (6), 2241–2250. doi: 10.1007/s13197-020-04260-5 32431350 PMC7230082

[B57] Ramirez J. Zambrano R. Sepúlveda B. Kennelly E. Simirgiotis M. (2015). Anthocyanins and antioxidant capacities of six Chilean berries by HPLC-HR-ESI-ToF-MS. Food Chem. 176, 106–114. doi: 10.1016/j.foodchem.2014.12.039 25624212

[B58] Reyes A. Evseev A. Mahn A. Bubnovich V. Bustos R. Scheuermann E. (2011). Effect of operating conditions in freeze-drying on the nutritional properties of blueberries. Int. J. Food Sci. Nutr. 62, 303–306. doi: 10.3109/09637486.2010.534078 21214411

[B59] Rodríguez K. Ah-Hen K. Vega-Gálvez A. López J. Quispe-Fuentes I. Lemus-Mondaca R.. (2014). Changes in bioactive compounds and antioxidant activity during convective drying of murta (*Ugni molinae* t.) berries. Int. J. Food Sci. Technol. 49, 990–1000. doi: 10.1111/ijfs.12392

[B60] Rodríguez K. Ah-Hen K. S. Vega-Gálvez A. Vásquez V. Quispe-Fuentes I. Rojas P.. (2016). Changes in bioactive components and antioxidant capacity of maqui, *Aristotelia chilensis* [Mol] stuntz, berries during drying. LWT - Food Sci. Technol. 65, 537–542. doi: 10.1016/j.lwt.2015.08.050

[B61] Ruiz A. Hermosín-Gutiérrez I. Mardones C. Vergara C. Herlitz E. Vega M.. (2010). Polyphenols and antioxidant activity of calafate (*Berberis microphylla*) fruits and other native berries from southern Chile. J. Agric. Food Chem. 58, 6081–6089. doi: 10.1021/jf100173x 20438111

[B62] Schaich K. M. Tian X. Xie J. (2015). Hurdles and pitfalls in measuring antioxidant efficacy: a critical evaluation of ABTS, DPPH, and ORAC assays. J. Funct. Foods 14, 111–125. doi: 10.1016/j.jff.2015.01.043

[B63] Scheuermann E. Seguel I. Montenegro A. Bustos R. Hormazábal E. Quiroz A. (2008). Evolution of aroma compounds of murtilla fruits (*Ugni molinae* turcz) during storage. J. Sci. Food Agric. 88, 485–492. doi: 10.1002/jsfa.3111

[B64] Schmeda-Hirschmann G. Delporte C. Valenzuela-Barra G. Silva X. Vargas-Arana G. Lima B.. (2014). Anti-inflammatory activity of animal oils from the *Peruvian amazon.* J. Ethnopharmacol 156, 9–15. doi: 10.1016/j.jep.2014.08.010 25150527

[B65] Sheng Z. Z. (2020). Anticancer effects of catechin flavonoid in human glioma cells are mediated *via* autophagy induction, cell cycle arrest, inhibition of cell migration and invasion and targeting MAPK/ ERK signalling pathway. J. B.U.ON. 25, 1084–1090. 32521910

[B66] Speisky H. Shahidi F. de Camargo A. C. Fuentes J. (2022). Revisiting the oxidation of flavonoids: loss, conservation or enhancement of their antioxidant properties. Antioxidants 11, 1–28. doi: 10.3390/antiox11010133 PMC8772813 35052636

[B67] Sun Y. Zhang M. Mujumdar A. (2019). Berry drying: mechanism, pretreatment, drying technology, nutrient preservation, and mathematical models. Food Eng. Rev. 11, 61–77. doi: 10.1007/s12393-019-9188-3

[B68] Tabart J. Franck T. Kevers C. Pincemail J. Serteyn D. Defraigne J. O.. (2012). Antioxidant and anti-inflammatory activities of ribes nigrum extracts. Food Chem. 131, 1116–1122. doi: 10.1016/j.foodchem.2011.09.076

[B69] Vega-Gálvez A. Díaz R. López J. Galotto M. J. Reyes J. E. Perez-Won M.. (2016). Assessment of quality parameters and microbial characteristics of cape gooseberry pulp (*Physalis peruviana* l.) subjected to high hydrostatic pressure treatment. Food Bioprod. Process. 97, 30–40. doi: 10.1016/j.fbp.2015.09.008

[B70] Vega-Gálvez A. Uribe E. Pastén A. Vega M. Poblete J. Bilbao-Sainz C.. (2022). Low-temperature vacuum drying as novel process to improve papaya (*Vasconcellea pubescens*) nutritional-functional properties. Futur. Foods 5, 0–7. doi: 10.1016/j.fufo.2022.100117

[B71] Wojdyło A. Figiel A. Legua P. Lech K. Carbonell-Barrachina Á.A. Hernández F. (2016). Chemical composition, antioxidant capacity, and sensory quality of dried jujube fruits as affected by cultivar and drying method. Food Chem. 207, 170–179. doi: 10.1016/j.foodchem.2016.03.099 27080894

[B72] Woo K. S. Kim H. Y. Hwang I. G. Lee S. H. Jeong H. S. (2015). Characteristics of the thermal degradation of glucose and maltose solutions. Prev. Nutr. Food Sci. 20, 102–109. doi: 10.3746/pnf.2015.20.2.102 26175997 PMC4500512

[B73] Zhang L. Li J. Hogan S. Chung H. Welbaum G. E. Zhou K. (2010). Inhibitory effect of raspberries on starch digestive enzyme and their antioxidant properties and phenolic composition. Food Chem. 119, 592–599. doi: 10.1016/j.foodchem.2009.06.063

[B74] Zhao G. Zhang R. Liu L. Deng Y. Wei Z. Zhang Y.. (2017). Different thermal drying methods affect the phenolic profiles, their bioaccessibility and antioxidant activity in *Rhodomyrtus tomentosa* (Ait.) hassk berries. LWT - Food Sci. Technol. 79, 260–266. doi: 10.1016/j.lwt.2017.01.039

[B75] Zia M. P. Alibas I. (2021). Influence of the drying methods on color, vitamin c, anthocyanin, phenolic compounds, antioxidant activity, and *in vitro* bioaccessibility of blueberry fruits. Food Biosci. 42, 101179. doi: 10.1016/j.fbio.2021.101179

[B76] Zielinska M. Zielinska D. Markowski M. (2018). The effect of microwave-vacuum pretreatment on the drying kinetics, color and the content of bioactive compounds in osmo-Microwave-Vacuum dried cranberries (*Vaccinium macrocarpon*). Food Bioprocess Technol. 11, 585–602. doi: 10.1007/s11947-017-2034-9

